# Self-administered questionnaires enhance emotion estimation of individuals with autism spectrum disorders in a robotic interview setting

**DOI:** 10.3389/fpsyt.2024.1249000

**Published:** 2024-02-06

**Authors:** Shunta Konishi, Masaki Kuwata, Yoshio Matsumoto, Yuichiro Yoshikawa, Keiji Takata, Hideyuki Haraguchi, Azusa Kudo, Hiroshi Ishiguro, Hirokazu Kumazaki

**Affiliations:** ^1^ Department of Human and Engineered Environmental Studies, Graduate School of Frontier Sciences, The University of Tokyo, Chiba, Japan; ^2^ Department of Medical and Robotic Engineering Design, Faculty of Advanced Engineering, Tokyo University of Science, Tokyo, Japan; ^3^ Department of Systems Innovation, Graduate School of Engineering Science, Osaka University, Osaka, Japan; ^4^ National Center of Neurology and Psychiatry, Department of Preventive Intervention for Psychiatric Disorders, National Institute of Mental Health, Tokyo, Japan; ^5^ Department of Neuropsychiatry, Graduate School of Biomedical Sciences, Nagasaki University, Nagasaki, Japan; ^6^ College of Science and Engineering, Kanazawa University, Kanazawa, Japan

**Keywords:** autism spectrum disorders, machine learning, self-administered questionnaire, affective state, automated estimation

## Abstract

**Background:**

Robots offer many unique opportunities for helping individuals with autism spectrum disorders (ASD). Determining the optimal motion of robots when interacting with individuals with ASD is important for achieving more natural human-robot interactions and for exploiting the full potential of robotic interventions. Most prior studies have used supervised machine learning (ML) of user behavioral data to enable robot perception of affective states (i.e., arousal and valence) and engagement. It has previously been suggested that including personal demographic information in the identification of individuals with ASD is important for developing an automated system to perceive individual affective states and engagement. In this study, we hypothesized that assessing self-administered questionnaire data would contribute to the development of an automated estimation of the affective state and engagement when individuals with ASD are interviewed by an Android robot, which will be linked to implementing long-term interventions and maintaining the motivation of participants.

**Methods:**

Participants sat across a table from an android robot that played the role of the interviewer. Each participant underwent a mock job interview. Twenty-five participants with ASD (males 22, females 3, average chronological age = 22.8, average IQ = 94.04) completed the experiment. We collected multimodal data (i.e., audio, motion, gaze, and self-administered questionnaire data) to train a model to correctly classify the state of individuals with ASD when interviewed by an android robot. We demonstrated the technical feasibility of using ML to enable robot perception of affect and engagement of individuals with ASD based on multimodal data.

**Results:**

For arousal and engagement, the area under the curve (AUC) values of the model estimates and expert coding were relatively high. Overall, the AUC values of arousal, valence, and engagement were improved by including self-administered questionnaire data in the classification.

**Discussion:**

These findings support the hypothesis that assessing self-administered questionnaire data contributes to the development of an automated estimation of an individual’s affective state and engagement. Given the efficacy of including self-administered questionnaire data, future studies should confirm the effectiveness of such long-term intervention with a robot to maintain participants’ motivation based on the proposed method of emotion estimation.

## 1 Introduction

Autism spectrum disorders (ASD) are a set of diverse neurodevelopment disorders characterized by difficulty with social interactions and behavioral difficulties. Individuals with ASD may behave, communicate, interact, and learn in ways that are different from most other people. The estimated 5-year lifetime cumulative incidence of ASD in children born between 2009 and 2014 in Japan is 2.75% (1). Regarding the economic impact, the cost of supporting an individual with ASD is expensive (e.g., his or her lifetime is estimated over $3.6 million in USA) (2). There are a variety of interventions for ASD. Many individuals with ASD cannot easily sustain high motivation and concentration in human interventions (3). Indeed, the dynamic facial features and expressions of humans may induce sensory and emotional overload and distraction (4). This overload can hamper interactions, as these individuals tend to actively avoid sensory stimulation and instead focus on more predictable elementary features. In addition, individuals with ASD struggle to generalize skills learned in intervention to everyday use, which is one of the greatest barriers to intervention success (5–7).

There is increasing anecdotal and case-based evidence that robots can offer many unique opportunities for individuals with ASD to learn social skills (8–12). It is also known that Android robots, whose appearances and movements resemble those of humans, can greatly promote social skill learning (13–18). Additionally, android robots exhibit various facial expressions (e.g., smiling, nodding, and brow movements) during speech and can provide subtle nonverbal cues. Therefore, generating intelligent three-dimensional learning environments using android robots may represent a powerful avenue for enhancing skills with generalization to real-world settings. Previous studies (13, 14) using android robot in a mock job interview setting showed skill enhancement with generalization to the real-world settings.

If a patient does not develop a positive attitude toward the robot, the intervention becomes challenging. As each individual with ASD has strong likes and dislikes (19), the optimal motion of android robots for facilitating communication differs among these individuals (20). However, in previous studies using android robots, the android parameters were uniform rather than personalized (13–18). In addition, the emotions and attention of the individuals with ASD may easily change during the intervention (21).

Moreover, many individuals with ASD have atypical and diverse ways of expressing their affective-cognitive states (22, 23). To address their heterogeneity, elucidating the optimal motion of robots for a given individual considering their traits and state is important for achieving more natural human-robot interactions and for exploiting the full potential of robotic interventions.

To achieve optimal robot motion for individuals with ASD, it is important to develop autonomous robots that can learn and recognize behavioral cues and respond smoothly to an individual’s real-time state (24). Most prior studies have focused on applying supervised machine learning (ML) to enable robot perception of user engagement directly from user behavioral data (e.g., child vocalizations, facial and body expressions, and physiological data such as the heart rate) (25–27). However, these studies have struggled to develop computational models to estimate an individual’s state from behavioral data, owing to the lack of consideration of individuals’ ASD symptoms.

A previous study demonstrated the technical feasibility of considering an individual’s symptoms when using ML to enable robot perception of affect and engagement in individuals with ASD (28). In this study, the Childhood Autism Rating Scale (CARS) (29) was used to assess the presence and severity of symptoms of ASD. The study revealed that the expert assessment provided by CARS data (29) improved estimation of the state of individuals with ASD when interacting with robots. Their idea of including personal demographic information in the estimation of the state of individuals with ASD is important for developing an automatic system that can perceive individual affective states and engagement. On the other hand, the CARS takes a long time to administer, and specialists (and special training) are needed to conduct the CARS.

Self-administered questionnaires are known to have several limitations, including insufficient objectivity; however, they do not require the involvement of trained clinicians and can be completed rapidly. In clinical situations, self-administered questionnaires, such as the Autism Spectrum Quotient-Japanese version (AQ-J (30);), Adolescent/Adult Sensory Profile (AASP;^31^), and LSAS (32), can provide therapists with valuable information on individuals with ASD. Ease of obtaining personal demographic information is important when developing computational models for individuals with ASD. We hypothesized that assessing self-administered questionnaire data contributes to the development of an automated estimation of affective state and engagement when individuals with ASD are interviewed by an Android robot, which will be linked to implementing long-term interventions and maintaining participant motivation.

Automated estimation of an individual’s affective states and engagement is considered important for long-term intervention, as the states and engagement of participants can change daily and/or dynamically during the interaction. This study therefore aimed to assess the contribution of self-administered questionnaire data to develop an automated estimation of an individual’s affective states and engagement when individuals with ASD were interviewed by an android robot. The goal of the current study was to obtain an accurate emotion estimator for individuals during interactions by maximizing the area under the curve (AUC) values, a conventional measure used in the field of machine learning.

## 2 Materials and methods

### 2.1 Samples

#### 2.1.1 Population

The inclusion criteria for individuals with ASD were as follows: 1) diagnosed with ASD based on the Diagnostic and Statistical Manual of Mental Disorders, fifth edition (DSM-5), by the supervising study psychiatrist, and 2) not currently taking medication. Thirty-five individuals with ASD participated in this study. Instead of adopting a standard statistical test paradigm with power analysis using G*Power, we adopted a five-fold cross-validation test paradigm to evaluate the goodness of fit (AUC) of the model, which is commonly used in machine learning (33). To determine the number of samples used for model fitting, we collected a similar number of samples [35] to that reported by Rudovic (28). Three participants lost concentration during the experiment and were unable to complete the experiment. Seven participants were unable to correctly perform gaze calibration. Finally, 25 samples (males 22, females 3, average chronological age = 22.8, average IQ = 94.04) completed the experiment without any technical challenges or distress that would have led to the termination of the session. All samples had no regular jobs. Details are presented in **Table 1**. At the time of enrollment, the diagnoses of all participants were confirmed by a psychiatrist with more than fifteen years of experience in ASD using standardized criteria taken from the Diagnostic Interview for Social and Communication Disorders (DISCO) (34). The DISCO has good psychometric properties (35). To exclude other psychiatric diagnoses, the Mini-International Neuropsychiatric Interview (MINI) (36) was administered.

**Table 1:** Descriptive statistics of participants.

	(M, SD)
Age	22.80 (5.25)
Sex (male:female)	22:3
AQ-J score	26.36 (6.18)
Full-scale IQ	94.04 (11.06)
LSAS score	55.68 (28.51)
AASP score	
Low registration score	42.12 (18.12)
Sensation seeking score	37.92 (9.39)
Sensory sensitivity score	38.88 (7.92)
Sensation avoiding score	39.00 (10.18)

M, mean; SD, standard deviation.

AQ-J, Japanese version of the Autism Spectrum Quotient. Higher scores reflect a higher number of ASD-specific behaviors.

LSAS, Liebowitz Social Anxiety Scale.

AASP, Adolescent/Adult Sensory Profile.

#### 2.1.2 Ethical procedure

The present study was approved by the Ethics Committee of Kanazawa University and was conducted in accordance with the Declaration of Helsinki. The authors had no conflict of interest. Participants were recruited by flyers that explained the content of the experiment. After receiving a complete explanation of the study, all participants and their guardians agreed to participate in the study. Written informed consent was obtained from the individuals and/or the legal guardian (of minors) for the publication of any potentially identifiable images or data included in this article.

### 2.2 Self-Administered questionnaire

All participants completed the Autism Spectrum Quotient-Japanese version (AQ-J) (30), a self-administered questionnaire used to measure autistic traits and evaluate ASD-specific behaviors and symptoms. The AQ-J is a self-administered questionnaire with five subscales (social skills, attention switching, attention to detail, imagination, and communication). Previous work with the AQ-J has been replicated across cultures (37) and ages (38). The AQ is sensitive to the broader autism phenotype (39). In this study, we did not use the AQ-J score as a cutoff for ASD and used only the DSM-5 and DISCO to diagnose ASD and to determine whether to include participants in our study. It takes approximately 10 minutes to complete the AQ-J.

Full-scale IQ scores were obtained with the Japanese Adult Reading Test (JART), a standardized cognitive function test used to estimate the premorbid intelligence quotients (IQ) of examinees with cognitive impairments (40). The JART has good validity for measuring IQ. The JART results are comparable to those of the WAIS-III (40). It takes approximately 10 minutes to complete the JART. Usually, the JART is conducted in a face-to-face interview setting. The participant is instructed to read the characters. In this study, we prepared a self-administered version of the JART in which participants described the reading.

The severity of social anxiety symptoms was measured using the Liebowitz Social Anxiety Scale (LSAS) (32), a 24-item self-rated scale that measures the role of social phobia in life across various situations. This self-administered questionnaire included 13 items that relate to performance anxiety and 11 concern social interaction situations. Each item was separately rated in terms of “fear” and “avoidance” using a 4-point categorical scale. Therefore, there are 48-items in total. According to receiver-operating curve analyses, an LSAS score of 30 is correlated with minimal symptoms and is the best cutoff value for distinguishing individuals with and without social anxiety disorder (41). It takes approximately 10 minutes to complete the LSAS.

The Adolescent/Adult Sensory Profile (AASP) is a self-administered questionnaire that measures sensory processing in individuals aged 11 years and older (31). The internal consistency coefficients of the AASP range from 0.64 to 0.78 for the quadrant scores. In this study, before the experiment, the participants reported how often they exhibited certain behaviors related to sensory experiences on a scale of one (almost never) to five (almost always). The AASP examines four different “quadrants” of sensory processing: low registration, sensation seeking, sensory sensitivity, and sensation avoiding. As the AASP does not categorize responses according to perceptual domains (e.g., auditory, visual, tactile), a perceptual domain analysis was not performed in this study. This scale takes approximately 10 minutes to complete. Please see the details of feature set (i.e., self-administered questionnaire) in **Table 2**.

**Table 2:** Description of the behavioral data and self-administered questionnaire data.

Feature	Dimension	Description
Audio	88	Acoustic feature values of utterance analyzed by OpenSMILE: ZCR, HNR, F0, MFCC, etc. in the GeMAPS v01b feature set.
Gaze	2	Probabilities of gaze distribution on the android’s face and body area: *p_face_, p_body_*
Motion	16	Standard deviations of the x and y coordinates (*σ_x_*, *σ_y_*) of eight key points of upper body joints (nose, right/left eyes, right/left ears, neck, and right/left shoulders) in a 2D camera image.
Self-administered Questionnaire	7	Strength of tendency of those characteristics in the diagnosis of interpersonal communication disorder. AQ-J score: Evaluation of ASD-specific behaviors and symptoms obtained from fifty questions on the level of agreement of AQ-J (1 value ranging from 0 to 50) Full-scale IQ: Evaluation of cognitive function calculated from the number of correct answers for fifty quizzes (1 value ranging from 76 to 124) LSAS score: self-rated 24-item scale assessing the role of social phobia in life across different situations in 13 performance and 11 social interaction situations with two scores (“fear” and “avoidance”) on a 4-point categorical scale (1 value ranging from 0 to 144) AASP score: a self-rated measurement of sensory processing in four different “quadrants”: (1) low registration, (2) sensation seeking, (3) sensory sensitivity, and (4) sensation avoiding with sensory experiences, each of which is calculated by accumulating answer scores on a scale of one (almost never) to five (almost always) for 15 questions (4 values ranging from 15 to 75)

### 2.3 Interviewer robot system

**Figure 1:**
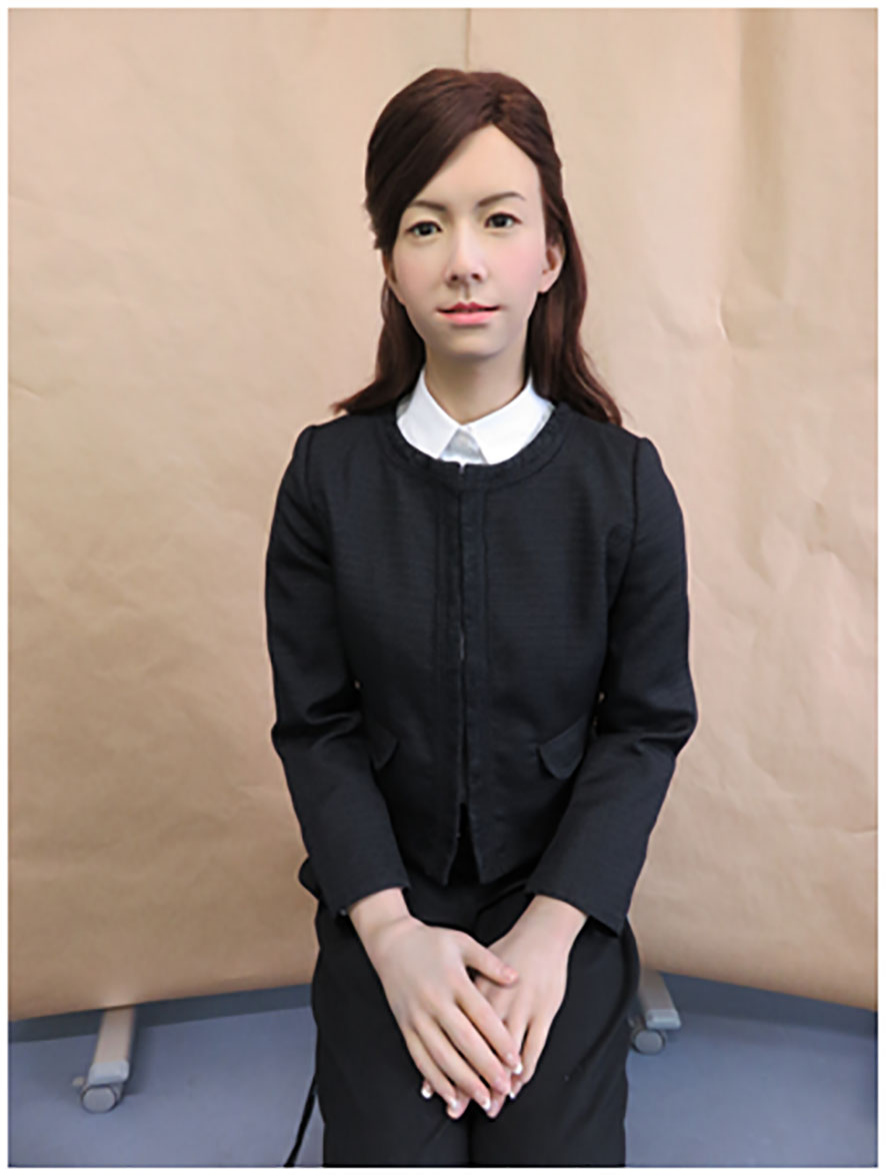
Android ST.

The robot used in this study was an Android ST by A-Lab Co., Ltd. (**Figure 1**), which is a female humanoid robot with an appearance similar to that of a real human. Its artificial body has the same proportions, facial features, hair color, and hairstyle as a human. The synthesized voice of the android is also similar to that of a human. To elicit the belief that the robot behaved and responded autonomously without any failures, we adopted a Wizard-of-Oz (WOz) method, similar to that conventionally used in robotics studies (42). Facial expressions (i.e., smiling, nodding, and brow movements) can be generated in addition to utterances during conversation.

### 2.4 Procedures

**Figure 2:**
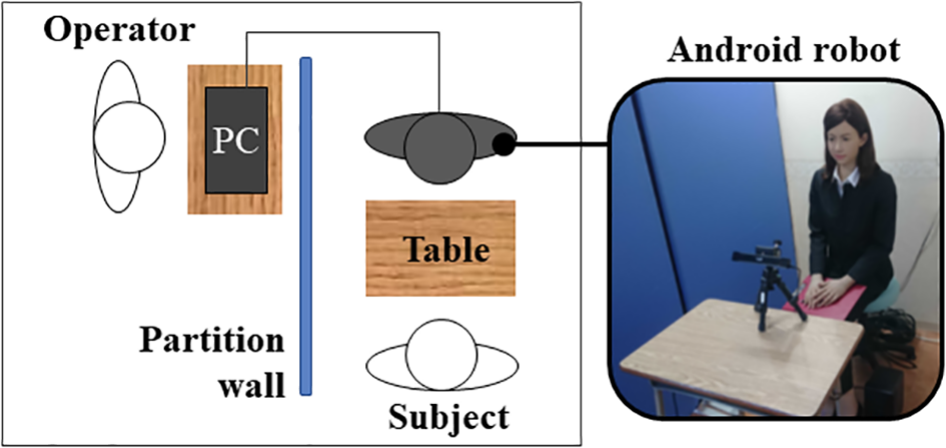
The experimental room.

In the experiment, a participant entered the room and sat across the table from the android, as shown in **Figure 2**. The gaze tracking device on the table was then calibrated to measure the participant’s gaze. In the beginning of the interaction with the android, the participant is asked to adjust the volume and the speed of the synthesized voice to comfortable levels by changing the parameters. There were 5 volume levels and 5 speed levels.

The android played the role of the interviewer, sitting in front of the participant, and the WOZ operator sat behind a partition, as shown in **Figure 2**. Each participant underwent four sessions of mock job interviews as the interviewee. Each session corresponded to one of the four conditions regarding android behavior, namely, with and without idle body motions and eye-blinking motions. The order of the four conditions was randomly assigned and counterbalanced among participants to reduce the order effect. The reason for including four conditions is that individuals with ASD vary regarding preferred behaviors (20).

In each interview session, question-and-answer conversations were conducted 9 or 10 times, initiated by the android interviewer. The android asked questions based on predefined sentence lists (see the **Supplementary Material**) and waited until the participant answers. This simple form of conversation was adopted to reduce variation in the dialog structure.

### 2.5 Behavioral measurement

**Figure 3:**
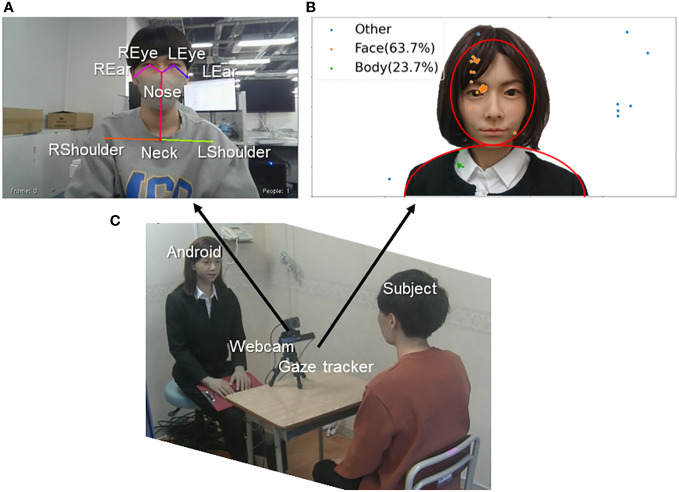
**(A)** Example gaze distributions of two-dimensional gaze information. **(B)** Example upper body posture for the calculation of motion data. **(C)** Overview of the experimental scene.

In each session, the behavior of the participant was recorded using a standard webcam (Logicool C980GR) and a gaze tracker (Tobii X2-30) installed on the desk as shown in **Figure 3C**. In each interview session, the participant answered the android interviewer 9 or 10 times. A set of behavioral data (audio, motion, and gaze) was collected that corresponded to each speech segment of the participant. Details of the measurement and feature extraction methods are described below. Please see the details of feature set (i.e., audio, motion, and gaze) in **Table 2**.

#### 25.1 Audio data

The audio data of the participant while interacting with the android robot were collected and stored in a computer and then analyzed afterward. For feature extraction, open-source speech and music interpretation by large-space extraction (OpenSMILE) (43) was adopted. It is a software toolkit for audio analysis, processing and classification, especially for speech and music applications. For the output of OpenSMILE, 88-dimensional feature values in the GeMAPS v01b feature set were utilized as previously described by Eyben et al. (44), including ZCR, HNR, F0, and MFCC. The zero crossing rate (ZCR) is the rate at which the sound waveform data cross zero in a given period. It is a key feature for classifying percussive sounds and tends to be higher for the speech part of the signal. The harmonics to noise ratio (HNR) is the ratio of harmonic and noise components that provides an indication of the general frequency of the speech signal by quantifying the relationship between the periodic component (harmonics) and the aperiodic component (noise). F0 is the fundamental frequency, and MFCC is the Mel frequency cepstral coefficient of the sound.

#### 2.5.2 Motion data

Video recordings of the upper body of subjects were collected with a webcam during the session. These data were converted into 16-dimensional motion feature data by image processing using OpenPose (45). It is an open-source library that uses part confidence maps to estimate the joint positions of the human body at high speed. In this study, eight key points on the upper body (nose, right/left eyes, right/left ears, neck, and right/left shoulders) were measured in 2-dimension (2D), as shown in **Figure 3A**. Subsequently, standard deviations of the 2D positions of the eight key points in a time series were calculated and used as 16-dimensional feature values, which indicated the degree of the participants’ movement/steadiness during each speech segment.

#### 2.5.3 Gaze data

The gaze data of the participant in the interaction were collected by a gaze tracker to determine how much the participant looked at the android. The system requires calibration before measurements can be collected. **Figure 3B** shows examples of the acquired 2D gaze distribution. Based on the gaze coordinates, the ratio of time elapsed while looking at the android’s face and body in each speech segment was calculated, yielding 2D feature values.

### 2.6 Annotation of emotional state

In this study, the emotional state of participants was represented based on Russell’s circumplex model (46). This model has the following three axes: 1) arousal, which is an indicator of concentration; 2) valence, which is an indicator of pleasure or displeasure; and 3) engagement, which is an indicator of interest. To apply machine learning techniques to estimate the emotional state of participants from the measured multimodal data, the ground truth emotion data were generated by manual annotation of the recorded video sequences. The methods and criteria of emotion annotation were established by an experienced psychologist who later instructed two research assistants in coding. The research assistants performed independent annotations and communication regarding the criteria until an intraclass correlation coefficient (ICC) of 0.8 or higher was obtained. The emotion annotations occurred only in the speech segments when the participant answered the robot interviewer. Therefore, the number of annotations was equal to the number of questions in the interview.

### 2.7 Data analysis

The collected behavioral data (audio, motion, and gaze) and the self-administered questionnaire data were used to train a model to estimate the emotional state of subjects. The training model was LightGBM (47). It is an ensemble machine learning algorithm that uses a gradient boosting framework based on decision trees (GBDT) and has faster training speed, lower memory usage, and better accuracy than other boosting algorithms. The binary classification model in Microsoft LightGBM v4.0.0, an open-source library for Python, was used in this study.

**Figure 4:**
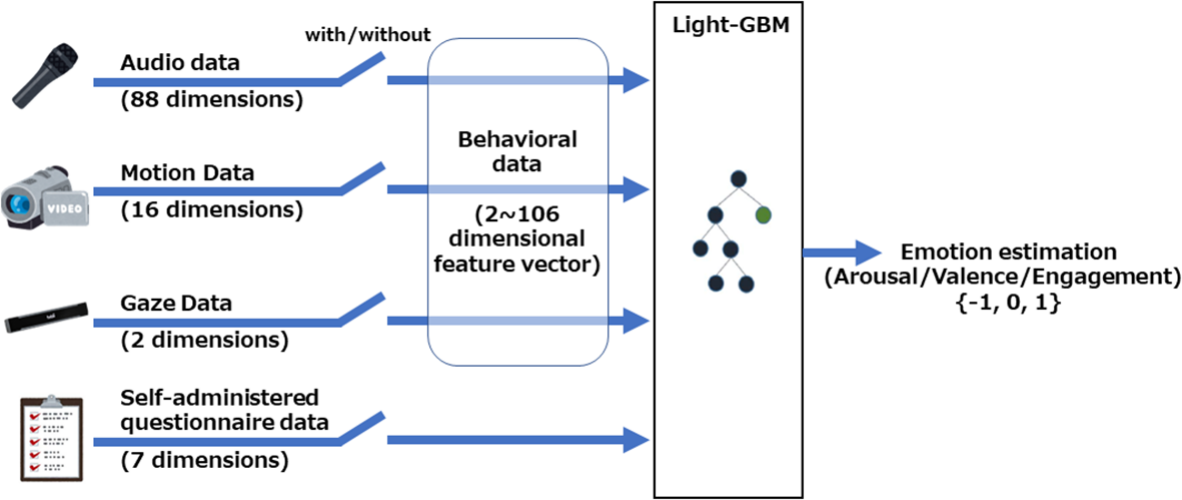
Overview of the automatic estimation system for determining individual emotional state based on machine learning. The collected behavioral data (audio, motion, and gaze) were used to train models with and without self-administered questionnaire data. LightGBM was utilized as the machine learning method.

We tested seven combinations of behavioral information (1) audio only, 2) motion only, 3) gaze only, 4) audio+motion, 5) audio+gaze, 6) motion+gaze, and 7) audio+motion+gaze) to investigate the importance of each type of behavioral data. We also tested the estimation with and without self-administered questionnaire data to investigate the importance of the self-administered questionnaire data (see **Figure 4**). The performance of the estimation model was evaluated based on the AUC of a fivefold cross-validation. The AUC, which is the area under the receiver operating characteristic (ROC) curve, is the measure of the ability of a binary classifier in machine learning to distinguish between two classes. The ROC curve was used to indicate the connection/trade-off between clinical sensitivity and specificity of the cutoff. The AUC has a value between 0 and 1, and higher AUC indicates better performance in distinguishing between classes.

## 3 Results

Participant performance was carefully monitored to ensure that all participants, except for three, were focused during the trial and remained highly motivated from the beginning to the end of the experiment. In total, 893 sets of feature vectors of behavioral measurements and annotated emotional states were obtained for 25 samples, which were then used to build the model for estimation.

**Figure 5:**
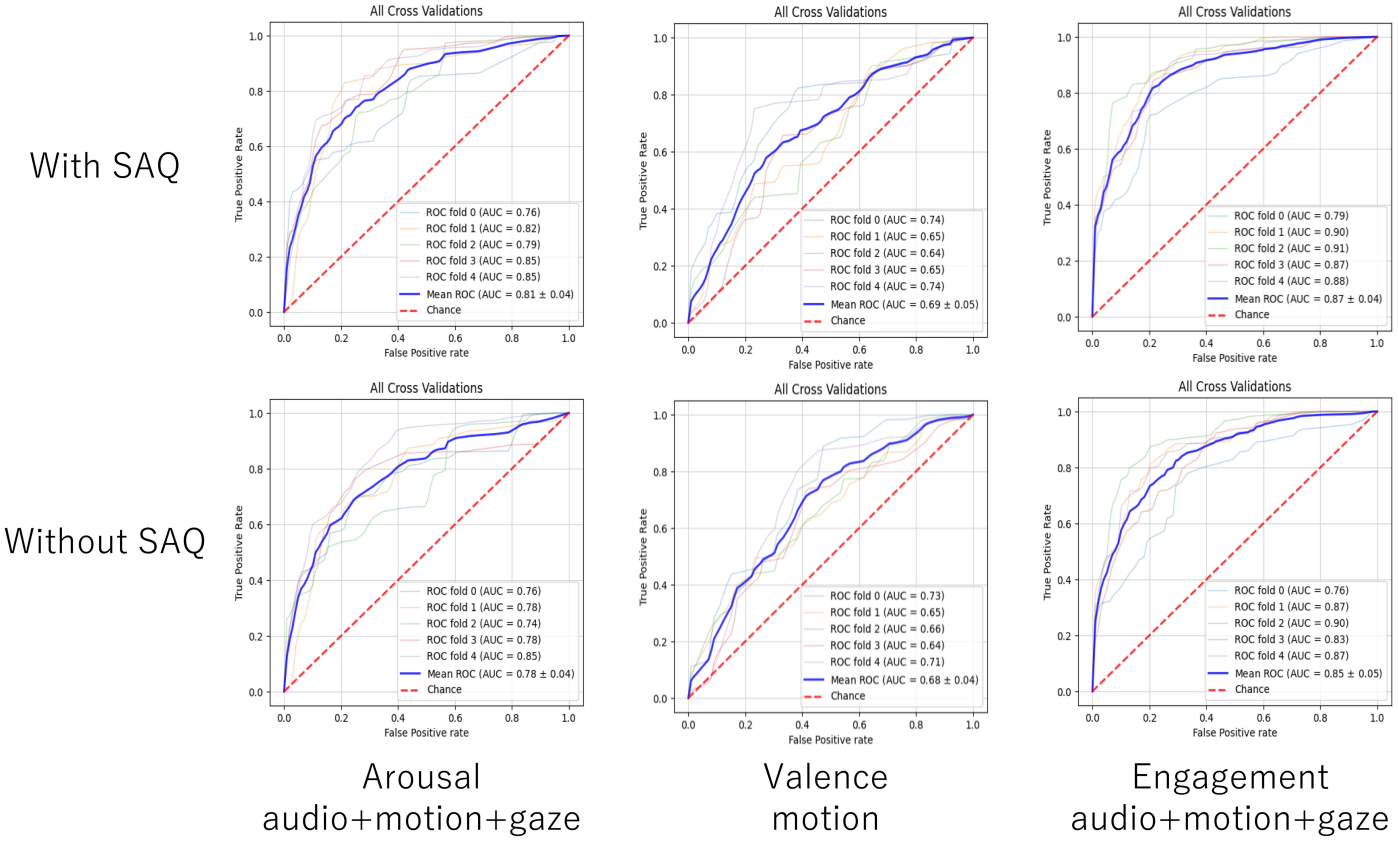
ROC curves showing the five validations and their average curve for the classifier with the highest AUC to classify arousal (left), valence (middle), and engagement (right) without (top) and with SAQ (bottom).

**Figure 6:**
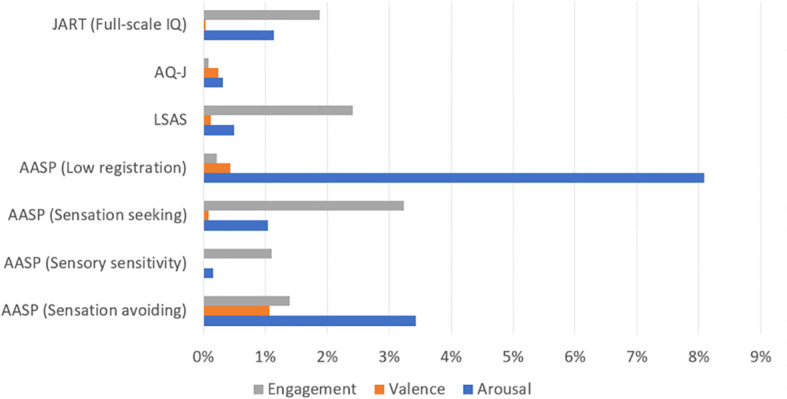
The contribution of self-administered questionnaire data to estimations of arousal, valence, and engagement (in terms of Gini importance) calculated with LightGBM, as shown in the “audio+motion+gaze with self-administered questionnaire” condition.

To evaluate the accuracy of the model based on the variables used, we adopted a five-fold cross-validation paradigm that is conventionally used in machine learning (36). **Table 3** shows the performance of emotion estimation models based on behavioral data. Each value indicates the AUC (mean and standard deviation) in the five-fold cross-validation test with and without self-administered questionnaire data. The graphs in **Figure 5** show the ROC curves for the five validations and the average curve for the classifier with the highest AUC to classify arousal, valence, and engagement with and without SAQ. **Figure 6** shows the contribution of each feature of the self-administered questionnaire data in terms of the Gini importance in the LightGBM.

**Table 3:** Estimation of emotions from behavioral data in terms of AUC.

	Arousal		Valence		Engagement	
	Without SAQ	With SAQ	Without SAQ	With SAQ	Without SAQ	With SAQ
Audio	0.72±0.06	0.77±0.06	0.63±0.05	0.62±0.05	0.82±0.05	0.85±0.05
Motion	0.71±0.05	0.80±0.05	0.68±0.04	0.69±0.05	0.68±0.05	0.78±0.04
Gaze	0.63±0.06	0.78±0.08	0.51±0.06	0.63±0.10	0.58±0.02	0.76±0.03
Audio+motion	0.78±0.03	0.81±0.02	0.66±0.05	0.69±0.05	0.85±0.04	0.87±0.03
Audio+gaze	0.73±0.06	0.78±0.06	0.62±0.03	0.63±0.04	0.83±0.05	0.85±0.05
Motion+gaze	0.75±0.06	0.81±0.04	0.66±0.04	0.67±0.06	0.70±0.06	0.78±0.04
Audio+motion+gaze	0.78±0.04	0.81±0.04	0.66±0.03	0.67±0.05	0.85±0.05	0.87±0.04

AUC, the area under the curve.

SAQ, self-administered questionnaire.

For arousal, in accordance with our hypothesis, the performance of the estimation model with self-administered questionnaire data was higher than that without self-administered questionnaire data for all combinations of behavioral data. The model with the highest AUC was audio+motion+gaze with self-administered questionnaire data (as shown in **Table 3**). Of the self-administered questionnaire variables, the low registration score had the highest contribution to estimations of arousal.

For valence, the AUC was higher when the self-administered questionnaire data were included for all combinations of the behavioral data except for the “audio only” case. The estimators with the highest AUC values were motion with self-administered questionnaire data, audio+motion with self-administered questionnaire data. Of the self-administered questionnaire variables, the sensory avoidance score had the highest contribution to estimations of valence.

For engagement, in accordance with our hypothesis, the AUC of the estimator was higher when the self-administered questionnaire data were included for all combinations of behavioral data. The estimators with the highest AUC values were audio+motion+gaze with self-administered questionnaire data and audio+motion with self-administered questionnaire data. Of the self-administered questionnaire variables, the sensory seeking score had the highest contribution to estimations of engagement.

## 4 Discussion

In this study, we assessed the contributions of self-administered questionnaire data to the development of an automatic system for detecting individual affective states and engagement during interviews with an android robot among individuals with ASD. Our experiments showed that leveraging the self-administered questionnaire data enhanced the emotional (i.e., arousal, valence, and engagement) estimation of individuals with ASD approximately by 0.02 on average in AUC. A previous study (28) that used the CARS also suggested that these data could facilitate the robot’s perception of affect and engagement in individuals with ASD. Previous work that focused on prediction, a different performance criterion, namely ICC, was used to verify accuracy. Therefore, it is not directly comparable with our study because it focused on the improvement of classification using the performance criterion of the AUC. However, the CARS requires more time (generally two to three hours, including the time needed for observation and filling out the score) than self-questionnaires (generally 40 minutes). In addition, the CARS requires the involvement of a specialist, whereas self-questionnaires do not need require such intervention. Therefore, the use of self-administered questionnaires may be more feasible for future implementation of robotic interventions.

In this study, among the self-administered questionnaire variables, the sensory profile would have a contribution to robot perception of affect and engagement. Kumazaki et al. (20) revealed that in an interview setting with an android robot, the sensory profile is an important factor for estimating the attitude of individuals with ASD toward android robots, which is in line with the results of this study.

Kim et al. (48) suggested that improving audio-based emotion estimation for individuals with ASD could allow the robotic system to properly assess the engagement of individuals, which is consistent with the results of this study. Rudovic et al. (28) previously suggested that audio data are insufficient to estimate a subject’s state, and that it is important to reduce background noise to utilize audio data. Unlike the previous study (28), in the present study, audio data would outperform the other single modalities in terms of the assessment of arousal and engagement, followed by a combination of motion and gaze data. This is considered to be caused by our experimental setup, where the environment was carefully prepared to be quiet, with reduced background noise.

Regarding the assessment of valence, motion data would outperform the other single modalities, followed by the combination of audio and gaze data. A previous study in the general population (49) suggested that motion is important for estimating valence, which is in line with the results of this study. Individuals with ASD exhibit abnormalities in posture (50) and coordination of balance (51). Given these factors, it is logical that motion data had important contributions to the estimation of the affective state of individuals with ASD in this study.

We showed that adding data from self-administered questionnaires would enable us to address heterogeneity in the representations of affective states and engagement in individuals with ASD (52). These results are linked to achieving more personalized and natural human-robot interactions and exploiting the full potential of robotic interventions.

This study had several limitations that should be addressed in future research. Firstly, the sample size was relatively small; therefore, future studies with larger sample sizes are warranted to validate our results. In this study, we only measured audio, motion, and gaze data in real time. However, monitoring other data, such as physiological data, may be useful to improve the AUC value. The number of self-administered questionnaires utilized in the study was also limited, meaning we were unable to fully capture the complete range of individual characteristics that influence affective states and engagement in individuals with ASD. Future studies including additional items are required to overcome these limitations. We discuss the data in light of ASD engagement behavior. Future studies should deliberate on ASD behaviors, such as empathy and nervousness. In addition, we conducted only semi-structured interviews. To create programs using Android robots that can be applied to a variety of situations, future studies with a variety of interview settings are required. Furthermore, the focus of this study on the interaction between individuals with ASD and an Android robot in a simulated job-interview setting may not mirror real-life social interactions entirely. Future studies investigating a variety of real-life social interactions are required. Our data showed that assessing self-administered questionnaire data contributes to the development of an automated estimation of an affective state and engagement when individuals with ASD are interviewed by android robots. Considering that the behavior of individuals with ASD toward robots is superior to humans (8–12), it is not clear whether the developed classifier works even for behavioral data obtained in the interaction with a human interviewer. In addition, we did not investigate whether the automated estimation of an individual’s affective state and engagement could be used to implement long-term interventions and maintain the motivation of participants. Future studies are required to ascertain the efficacy of data collection using self-administered questionnaires.

In the field of robotic interventions for individuals with ASD, few studies have demonstrated skill acquisition that is considered clinically meaningful and generalized beyond the specific robot encounter (53). To implement interventions with long-term effects and maintain the motivation of participants, it is important to develop technology to accurately and automatically perceive participant affect and engagement. The importance of adding data from self-administered questionnaires for emotion estimation reported in this paper could serve as a reference for the development of android robots that detect the effects and engagement of individuals, which would be the next step in establishing interventions involving android robots.

In this study, we assessed the contributions of self-administered questionnaire data to the development of an automatic system for detecting the affective states and engagement of individuals with ASD during interviews with an android robot. We leveraged self-administered questionnaire data and found that the estimation performance was enhanced for arousal, valence, and engagement. Our results also would support our hypothesis that the assessment of self-administered questionnaire data can contribute to the development of an automated estimation of an individual’s affective state and engagement when individuals with ASD are interviewed by an android robot. In the field of robotic interventions for individuals with ASD, few studies have demonstrated skill acquisition that is clinically meaningful and generalizable beyond the specific robot encounter. To implement long-term interventions and maintain the motivation of participants, technology that can automatically detect the affect and engagement of individuals in terms of personal traits is needed. Future studies should confirm the effectiveness of long-term intervention with a robot that can maintain the participants’ motivation based on the proposed method of emotion estimation.

## Data availability statement

The raw data supporting the conclusions of this article will be made available by the authors, without undue reservation.

## Ethics statement

The studies involving humans were approved by the Ethics Committee of Kanazawa University. The studies were conducted in accordance with the local legislation and institutional requirements. The participants provided their written informed consent to participate in this study. Written informed consent was obtained from the individuals and/or the legal guardian (of minors) for the publication of any potentially identifiable images or data included in this article.

## Author contributions

MK and HK designed the study, conducted the experiments, conducted the statistical analyses, analyzed and interpreted the data, and drafted the manuscript. MK, YM, YY, KT, HH, HI and HK, MK, and AK conceptualized the study, participated in its design, assisted with data collection and scoring of behavioral measures, analyzed and interpreted the data, drafted the manuscript, and critically revised the manuscript for important intellectual content. HK approved the final version to be published. All the authors have read and approved the final version of the manuscript.
