# Supplementary material for: Self-administered questionnaires enhance emotion estimation of individuals with autism spectrum disorders in a robotic interview setting
Source: Front Psychiatry. 2024 Feb 6;15:1249000. doi: 10.3389/fpsyt.2024.1249000 (PMC10877007; doi:10.3389/fpsyt.2024.1249000)
Supplement: Supplementary file 1 [file DataSheet_1.docx]

Supplementary Material

The dialog between the android interviewer and participant was partially structured by using the script presented below. The sentences listed in (1), (2), and (3) were the utterances of the android interviewer. The interviewer uttered a sentence from List 1, waited for a response from the participant, and then responded with a sentence from List 2. The utterances in List 3 were sometimes inserted to prompt the participant to respond to the theme more deeply. The sentences were presented in the order shown in List 1 on all days of the experiment, while one of the two sentences from List 3 was chosen for use on each day of the experiment.

(1) Script used for the mock online job interview:

1. Please take a seat.

2. Good afternoon. Hello there.

3. Thank you for applying to my company, [name].

4. Well then, could you introduce yourself?

5. Would you please tell me the reason that you applied?

6. Why do you want this job? Please tell me.

7. What are your strengths? What is your special talent?

8. What are your weaknesses?

9. How do you address these weaknesses?

10. What kind of things would you like to do at our company? Please tell me what you can do.

11. What are you doing in vocational training school?

12. Have you ever experienced a failure in your work?

13. Please explain your disability briefly.

14. How are you feeling now?

15. In my company, we have many jobs that require standing. Is that all right?

16. Are you able to carry heavy things?

17. Are you on any medication now?

18. Is there anything that you would like us to consider?

19. Which route did you take to come here from your home?

20. Are there any specific working days or hours that you prefer?

21. Is there anything else you would like to tell us?

22. I understand. Thank you very much.

(2) Words and phrases used as responses (to facilitate the conversation):

・ Yes

・ Certainly

・ All right

・ Very well

・ Thank you

・ I see. Now I understand.

・ Oh, I see. I got it.

・ I see. That makes sense.

・ Great!

・ Wonderful!

・ Fantastic!

・ Incredible!

(3) Phrases used to elicit further responses:

・ Please tell me a bit more in detail.

・ Why do you think so?
